# Role of Bcl-3 in solid tumors

**DOI:** 10.1186/1476-4598-10-152

**Published:** 2011-12-23

**Authors:** Vilma Maldonado, Jorge Melendez-Zajgla

**Affiliations:** 1 Instituto Nacional de Cancerología, Mexico City Mexico; 2 Institute of Genomic Medicine (INMEGEN), Periferico Sur 4124, Torre Zafiro II 5to piso, Col. Ex- Rancho de Anzaldo, Alvaro Obregon 01900, Mexico City, México

## Abstract

Bcl-3 is an established oncogene in hematologic malignancies, such as B-cell chronic lymphocytic leukemias. Nevertheless, recent research has shown that it also participates in progression of diverse solid tumors. The present review summarizes the current knowledge of Bcl3 role in solid tumors progression, including some new insights in its possible molecular mechanisms of action.

## Background

BCL3 was identified as a translocation into the immunoglobulin alpha-locus in several cases of B-cell chronic lymphocytic leukemias [1-4]. This oncogene is an atypical member of the Inhibitor of Kappa-B (IkappaB) family of proteins. Ikappa B proteins repress the activation of the NFkappa-B signaling cascade by direct binding to the dimeric transcription factors NFKB1, NFKB2, RELA, RELB or c-Rel. Interestingly, even with a high structural homology to the other family members [5,6], Bcl-3 is instead a nuclear protein with both transactivation and transrepressor functions [7-10]. These actions are mainly mediated by the formation of heterocomplexes with NFKB1 (p50) or NFKB2 (p52) homodimers, in which Bcl-3 provides two transactivating domains to the complex.

**Figure 1:**
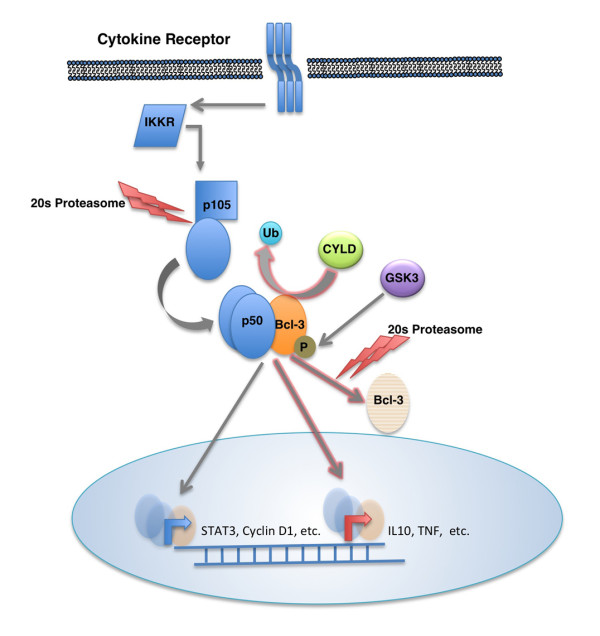
**Bcl-3 signaling cascade**. Diverse cytokines activate an IKK or IKK-related kinase to induce processing of the p105 precursor to p50, which in turn dimerizes and binds Bcl-3. Bcl-3 is regulated by phosphorylation and ubiquitination, both of which have a positive effect on its function. CYLD deubiquitinates Bcl-3 and prevents its nuclear translocation and GSK3. Bcl-3 is also phosphorylated by GSK3, which delays its degradation by the proteosomal pathway. Nuclear Bcl-3 can both induce and repress expression of a diverse array of genes.

Little is known about the physiological signaling cascades that activate Bcl-3. It has been reported that this oncogene is upregulated by several cytokines, including TNF alpha [11,12], IL-4 [13], IL-1 [14,15], IL-6 [16], IL-10 [17], adiponectin [18] and IL-12 [19]. These cytokines have in common their induction the activation of diverse signaling modules, such as AP1 [13] and STAT3 [16,20,21]. 'As with other members of the NF-kappa B family, Bcl-3 is regulated by NFKB1 and by itself, creating an autoregulatory loop to terminate its activation [16,22]. In addition, as described below, Bcl-3 is downregulated by p53 [23] (Figure 1).

Bcl-3 is also regulated by post-transcriptional mechanisms, such as translation [24,25] and protein stability [26-30]. Recently, in ovarian cancer cells, a new regulation step was found, which involves miR-125b a microRNA that decreased Bcl-3 translation [31], inhibiting tumor formation in nude mice. Less is known about the reported nuclear translocation regulation, in which the cylindromatosis gene product, CYLD, plays an important role in interleukin-mediated activation [15,32].

As stated previously, Bcl-3 forms a complex with p50 or p52 homodimers to regulate transcription. The first reports showed that Bcl-3 could be acting to enhance NF-kappa B-mediated transactivation by removing inhibitory p50 homodimers from NF-kappa consensus sites in diverse promoters [8,33]. Subsequent studies using better reagents, demonstrated that Bcl-3 could not dissociate p50 homodimers from promoters [9]. Instead, Bcl-3 can act as a coactivator for p50 and p52 dimers [7]. More recently, it has also been shown that, for other specific promoters, such as the TNF-alpha promoter, Bcl-3 is indeed able to inhibit NF-kappa B-mediated transactivation, by binding to p50 homodimers, without dissociating them from a promoter, but also without inducing transactivation [17,34,35].

**Figure 2:**
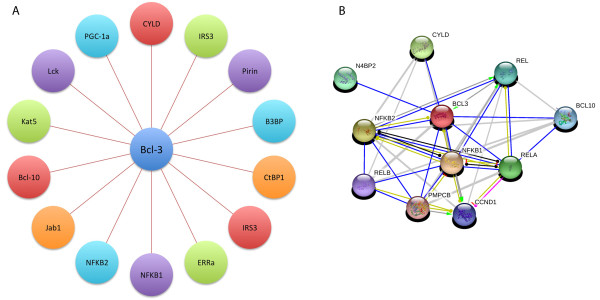
**A) Proteins that interact with Bcl-3**. B) Schematic representation of protein interactions from STRING (Search Tool for the Retrieval of Interacting Genes). Blue lines represent direct binding [79].

In addition to the gene products of the NF-kappa B signaling cascade, Bcl-3 also associates with several proteins such as Jab1, Pirin, Tip60 (KAT5) and Bard1, which are transcriptional co-regulators [36]. Bcl-2 also associates with B3BP, which is protein involved in DNA damage responses [37]; Lck, an important tyrosine kinase in hematological malignancies [38] and ERRalpha and PGC-1alpha, involved in metabolism [39]. Recent reports have shown that CtBP1, a transcriptional co-repressor [40]; IRS3, a substrate of insulin receptor and insulin-like growth factor (IGF)-I receptor tyrosine kinases [41] and Bcl-10 a CARD-containing protein that induces apoptosis [42] are also binding partners of Bcl-3 (Figure 2). These interactions and their physiological consequences have been little studied.

BCL3 locus has been found to be translocated not only in B-cell chronic leukemias, but also in other hematological malignancies, such as small lymphocytic lymphomas, Burkitt-like lymphoma and diffuse large cell lymphoma [43]. In addition, overexpression without translocation has been found in multiple subtypes of non-Hodgkin and Hodgkin lymphomas [44,45]. These results underscore the importance of this oncogene in hematological neoplasias. In addition to this, Bcl-3 has been found to be deregulated in breast cancer [46], nasopharyngeal carcinoma [47], endometrial cancer [48] and colorectal cancer [40]. Here, we present current knowledge of the role of this oncogene in solid tumor progression, including some new insights in its possible molecular mechanisms of action.

### Mechanism of deregulation

As with hematological malignancies, the most common Bcl-3 alteration found in solid tumors is overexpression. Nevertheless, in carcinomas, no translocations in the BCL3 locus have been found, pointing toward an activating upstream signal transduction cascade and/or epigenetic mechanism(s). Since NF-kappa B modulates BCL3 expression in an auto regulatory loop [22,35], and NF-kappa B is constitutively activated in several tumors [49-51], it is probably that the observed Bcl-3 deregulation could be due to aberrant NF-kappa B activation. Additionally, most of the studies rely solely in the amount of nuclear Bcl-3 as a surrogate marker for its activation, since there is no reliable specific DNA consensus-binding site for the oncogene. Since it has been shown that Bcl-3 is heavily phosphorylated and this phosphorylation modulates its activity [26], it remains an open question whereas lower Bcl-3 levels of a specific phosphoisoform could be also acting in cancer progression. As stated previously, recent reports have shown that cylindromatosis (CYLD) gene product regulates Bcl-3 [28,52]. The CYLD protein deubiquitinates Bcl-3 and inhibits its nuclear translocation, so alterations in these gene or upstream events to it present an additional layer of regulation.

It is important to note that the Catalog of Somatic Mutations in Cancer (COSMIC) [53] has a very low number of BCL3 mutations (2 missense mutations, one in a lung cancer and a second in an ovarian cancer patient) among its large database. These mutations most probably represent passenger mutations, since they apparently do not affect overall protein structure (additional file 1), and are not located in potential phosphorylation sites (not shown).

### Deregulation in Solid Tumors

Breast cancer was the first solid tumor in which evidence for Bcl-3 deregulation was found. Cogswell, et al demonstrated that Bcl-3 mRNA and protein is over expressed in breast tumors and cell lines [46]. Their results also suggested that NF-kappa B was active in the tissue, as genes regulated by this signaling cascade were also concomitantly regulated, in contrast to surrounding normal stroma. Supporting this finding is the report that transgenic mice overexpressing c-Rel under the strong promoter of mouse mammary tumor virus (MMTV) developed tumors that overexpressed p50, p52, RelA, RelB, and the Bcl-3 protein [54]. Additional evidence comes from animal studies, in which it has been shown that overexpression of Bcl-3 is able to increase the establishment and growth of breast cancer xenografts [55]. In addition, estrogen withdrawal in breast cancer cell lines increased the expression and activity of Bcl-3, providing an alternative proliferation pathway and further advantage for tumor growth in mice.

Bcl-3 is also overexpressed and activated in nasopharyngeal carcinomas [47], where it is bound to p50 homodimers. Among others, this complex is bound to the promoter of the receptor for the Epidermal Growth Factor, playing a crucial role in the overexpression of this oncogene [47,56,57]. Similar to this report, it has been shown that Bcl-3 is also overexpressed in endometrial tumors [48], where its nuclear expression correlates with p52 immunostaining. It is interesting to note that Bcl-3 was the NF-kappa B subunit most detected, with 62% of the patient samples presenting nuclear Bcl-3 protein.

Recently, the first report associating Bcl-3 expression with a clinical outcome has been published. Puvvada, et al demonstrated that nuclear immunostaining of Bcl-3 was strongly associated with survival, even more than the other NF-kappa B subunits analyzed in colorectal cancer. Using a weighted score that combines percentage of positive nuclei with staining intensity, these authors found a 91% increase in hazard for a death event for each 50-point increase in nuclear Bcl-3 expression [58].

It is interesting to note that, even with the evidence presented here, there is only one article [58] exploring the possible use of Bcl-3 as a diagnostic/prognostic factor. Clearly, more research in this area is needed. In this regard, it is interesting that, in addition to the known deregulation in leukemias and lymphomas, genome-wide expression studies have shown that Bcl-3 is overexpressed in breast cancer, glioblastoma tumors, ovarian cancer and, intriguingly, teratomas and embryonal carcinomas (additional file 2). Although not validated, these results support the potential importance of this oncogene in a variety of tumors.

### Mechanisms of oncogenesis

Two main effects of Bcl-3 on oncogenesis of solid tumors have been described: modulation of cell death and proliferation:

### Cell death

One initial discovery suggested that one of the main oncogenic effects of Bcl-3 in hematopoietic malignancies is to increase survival in a subset of cells. Transgenic mice overexpressing the oncogene developed splenomegaly and presented increased mature B cells in lymph nodes and bone marrow [59]. Similar results were obtained from *in vitro *studies with T cells, [60-63]. The expansion of these cell compartments could be due to a decrease in cell death, by means of apoptosis inhibition and an increase in proliferation, discussed below [63]. Diminished cell death and its consequent increased cell number could provide an advantage to survival and mutation accumulation, as demonstrated in other models [64].

Less studied is the role of Bcl-3 in apoptosis inhibition of solid tumors. Initial reports showed that Bcl-3-p52 dimers are able to transactivate the antiapoptotic gene BCL2 in MCF7AZ breast cancer cells [65]. More important, it has been shown that, in breast cancer cells, DNA damage up regulates Bcl-3, which induces the expression of HDM2, the main negative regulator of p53 [66]. p53 is a tumor suppressor gene that, in response to DNA damage, arrests cell cycle and induces apoptosis. HDM2 in turn, inhibits both expression and activity of p53 [67]. It has also been shown in a variety of cancer cells that basal apoptosis is suppressed by Bcl-3 in a regulatory loop induced by JNK1 and suppressed by the related JNK2 [68]. It is interesting to note that this loop is not involved in suppressing basal cell apoptosis in non-cancerous cells, pointing toward a possible "gene addiction" role of Bcl-3. More recently, a new interaction partner of Bcl-3, CtBP1, was found in breast cancer cells [40]. Bcl-3 stabilizes CtBP1 by blocking its degradation by the proteasome and inhibiting apoptosis, leading to the sustained repression of pro-apoptotic gene expression and subsequent inhibition of apoptosis. Interestingly, expression of Bcl-3 and CtBP1 is strongly correlated in breast cancer samples.

Recently, we have reported that Bcl-3 is involved in an additional death pathway that is independent of apoptosis [69]. Cervical cell lines in which Bcl-3 is knocked-down by a specific shRNA arrested temporally in G2/M, presented a DNA damage response and enter unsuccessful mitosis cycles which ultimately leads to centrosome amplification, increased aneuploidy, leading to a clonogenic death. These results could imply that Bcl-3 participates in an oncogene addiction phenomenon, in which inactivation of this gene would specifically kill cancer cells overexpressing Bcl-3, as reported for other genes [70]. Further research is needed to elucidate the exact molecular basis for this response, since p53 is already downregulated in these cells by human papillomavirus E6 protein [71] and thus, an additional mechanism could be expected. In this regard, we have found that Bcl-3 regulates STAT3 in cervical cancer cells [20]. STAT3 is an important oncogene in solid tumors that, among other functions, regulates the DNA damage response [72-74]. It has been reported that Bcl-3 is induced by activation of STAT3 due to Epstein-Barr LMP1 oncoprotein [21] and also by granulocyte colony-stimulating factor [75]. On the other hand, we have shown that Bcl-3 depletion decreases STAT3 expression [69]. Since both genes are regulated by each other [21,75], DNA damage may create an amplification loop that could be necessary for a correct cellular response. Forced expression or concomitant inhibition of both genes should provide an answer to this question.

### Proliferation

The first evidence for the effects in cell growth came from the previously mentioned transgenic mice overexpressing Bcl-3 [59]. These finding were extended to hematological malignancies, such as multiple myeloma [12]. More evidence of effects in proliferation is known from solid tumor models. Westerheide, et al demonstrated in breast cancer cell lines that Bcl-3, acting as a coactivator of p52 dimers, induced the expression of cyclin D1, and thus, increased the transition at G1/S cell cycle phase [76]. In turn, it has been shown that p53 decreased the expression of Bcl-3 changing p52/Bcl-3 to p52/HDAC complexes in the cyclin D1 promoter, thus inhibiting cyclin expression [23]. In the skin, Bcl-3 participates in a signaling module downstream of CYLD [28]. CYLD is mutated in the human syndrome cylindromatosis, in which affected patients present benign tumors in skin adnexa. In these cases, mutated CYLD is unable to deubiquitinate Bcl-3, allowing increased proliferation in cell of the skin adnexa [28]. Adding to this, it has been shown that CYLD is downregulated by Snail in malignant melanomas [77]. This downregulation allowed the stabilization, nuclear localization and transcriptional activation of Bcl-3, enhancing proliferation and invasion of these cells. Finally, the role of Bcl-3 in skin/adnexal tumorigenesis is also supported by a mouse model of skin carcinogenesis in which Bcl-3 is strongly overexpressed in late papillomas and squamous cell carcinoma [78].

## Conclusion

Bcl-3 is an established oncogene in hematologic malignancies, such as B-cell chronic lymphocytic leukemias. Nevertheless, recent research has shown that it also participates in progression of diverse solid tumors. As more information is available, it is clear that Bcl-3 is involved in central oncogenic pathways that regulate cell death and apoptosis, so it could be important as a target to validation as a diagnostic or prognostic marker in these tumors.

## List of abbreviations

Bcl-3: B-Cell Lymphoma 3; NFkappa B: Nuclear factor kappa-light-chain-enhancer of activated B cells; TNF-α: Tumor necrosis factor-α; Lck: lymphocyte-specific protein tyrosine kinase; Jab1: Jun Activation Domain Binding Protein 1; Pirin: iron-binding nuclear protein; KAT5: K(lysine) acetyltransferase 5; Bard1: BRCA1 associated RING domain 1; B3BP: Bcl-3 Binding Protein; STAT3: signal transducer and activator of transcription; IL1-12: Interleukin 1 to 12; ERRalpha: estrogen-related receptor alpha; PGC-1a: Peroxisome proliferator-activated receptor gamma coactivator 1-alpha; CYLD: cylindromatosis (turban tumor síndrome); CtBP1: C-terminal-binding protein 1; IRS3: insulin receptor substrate 3; GSK3: Glycogen synthase kinase 3.

## Competing interests

The authors declare that they have no competing interests.

## Authors' contributions

JM-Z wrote the first draft, VM contributed with specific sections, JM-Z and VM reviewed the manuscript and wrote the final version. Both authors read and approved the final manuscript.

## Supplementary Material

Additional file 1 **Structure prediction of mutated Bcl-3 proteins found in COSMIC database**. Bcl-3 sequence was retrieved from NCBI and mutation data from COSMIC. Proteins were modeled using SWISS-MODEL structure-homology server [80]. A) Bcl-3 wild type structure B) Bcl-3 pP420A mutant from a lung cancer sample C) Bcl-3 p.R145W mutant from an ovary cancer sample. Click here for file

Additional file 2 **Overexpression of Bcl-3 in different tumor types**. Oncomine™ (Compendia Bioscience, Ann Arbor, MI) Expression Arrays Database was used for analysis and visualization. A) Overexpression in Glioblastoma (180 samples) from Su, et. al.[81]. P-value in T test 1.44^-9 ^with a fold change of 3.307. Light blue (number 1) represents normal brain controls. Dark blue (number 2) are cancer samples. B) Overexpression in Breast Cancer from Finak, et. al. [82] (59 samples). P-value in T test 7.10^-15 ^with a fold change of 2.266. Light blue (number 1) represents normal breast controls. Dark blue (number 2) are cancer samples. C) Overexpression in ovarian cancer (32 samples) from Welsh, et. al. [83]. P-value in T test 6.04^-8 ^with a fold change of 23.955. Light blue (number 1) represents normal ovary controls. Dark blue (number 2) are cancer samples. D) Overexpression in teratomas from Korkola, et. al. [84] (20 samples). P-value in T test 6.64^-10 ^with a fold change of 11.591. Light blue (number 1) represents normal testis controls. Dark blue (number 2) are cancer samples. E) Overexpression in embryonal carcinomas (21 samples) from Korkola, et. al. [84]. P-value in T test 2.22^-6 ^with a fold change of 2.295. Light blue (number 1) represents normal testis controls. Dark blue (number 2) are cancer samples. Click here for file
