# Supplementary figures and images for: Role of Bcl-3 in solid tumors
Source: Mol Cancer. 2011 Dec 23;10:152. doi: 10.1186/1476-4598-10-152 (PMC3258214; doi:10.1186/1476-4598-10-152)

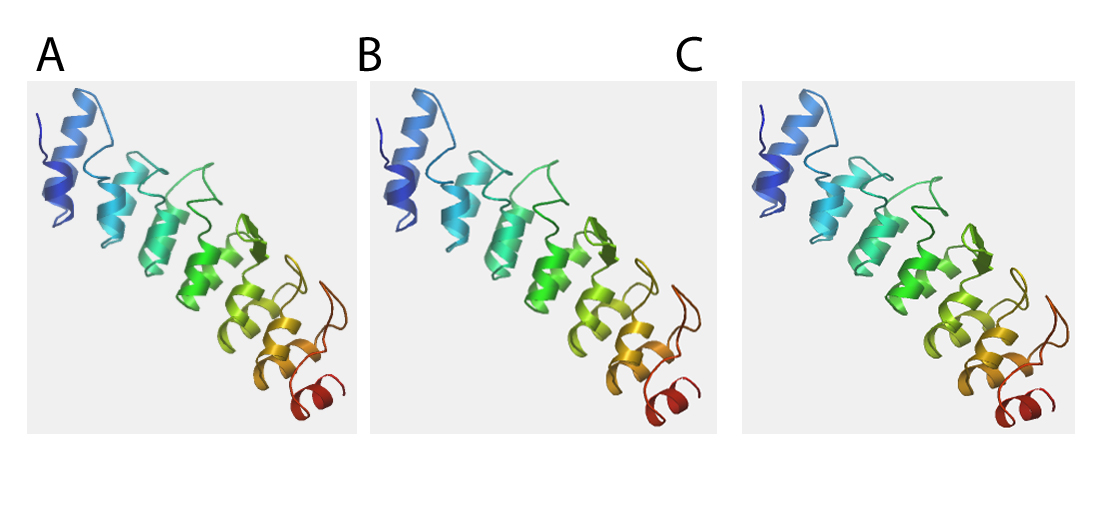

Supplement: Additional file 1 — Structure prediction of mutated Bcl-3 proteins found in COSMIC database. Bcl-3 sequence was retrieved from NCBI and mutation data from COSMIC. Proteins were modeled using SWISS-MODEL structure-homology server [80]. A) Bcl-3 wild type structure B) Bcl-3 pP420A mutant from a lung cancer sample C) Bcl-3 p.R145W mutant from an ovary cancer sample. [file 1476-4598-10-152-S1.JPEG]

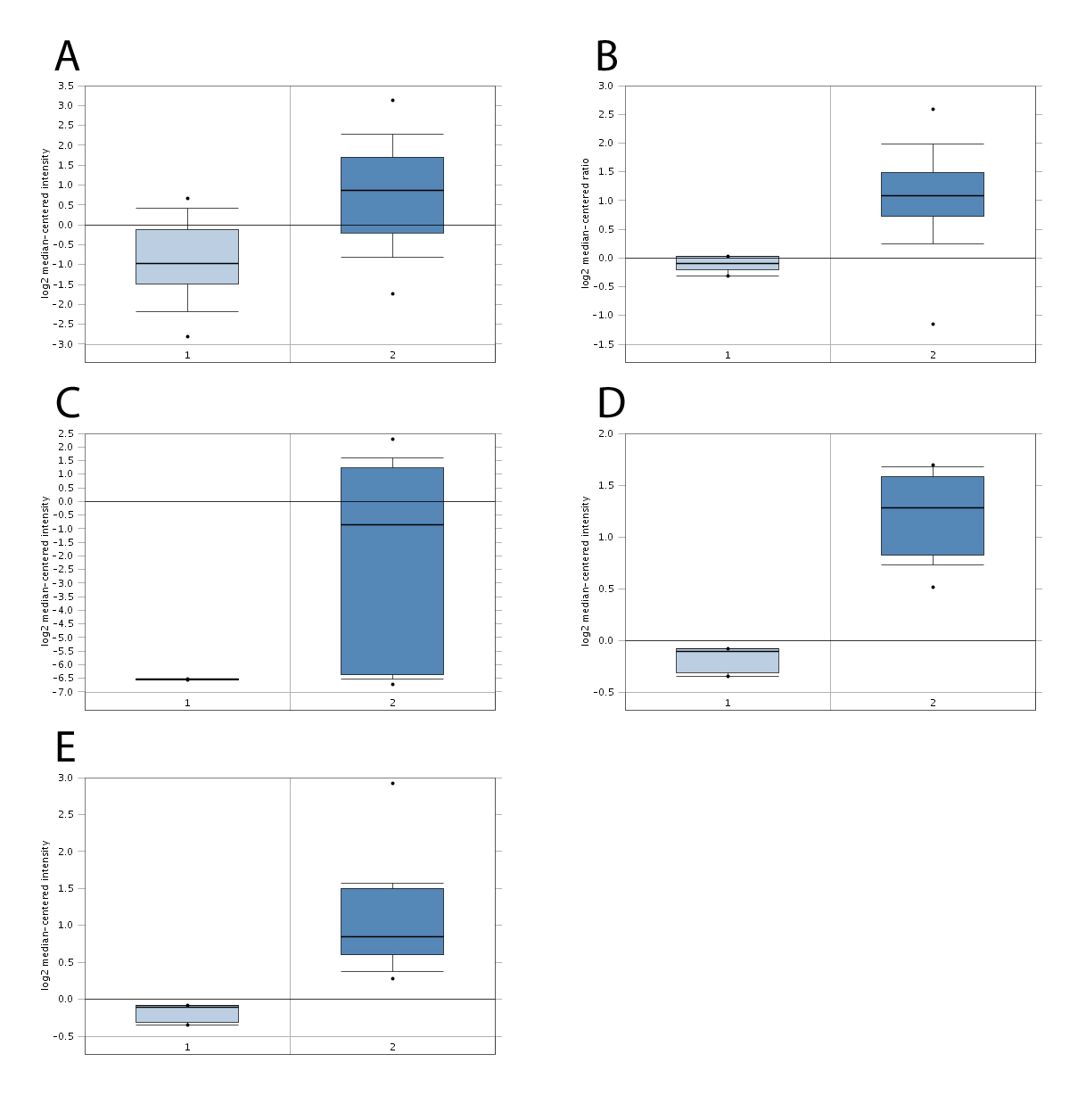

Supplement: Additional file 2 — Overexpression of Bcl-3 in different tumor types. Oncomine™ (Compendia Bioscience, Ann Arbor, MI) Expression Arrays Database was used for analysis and visualization. A) Overexpression in Glioblastoma (180 samples) from Su, et. al.[81]. P-value in T test 1.44-9 with a fold change of 3.307. Light blue (number 1) represents normal brain controls. Dark blue (number 2) are cancer samples. B) Overexpression in Breast Cancer from Finak, et. al. [82] (59 samples). P-value in T test 7.10-15 with a fold change of 2.266. Light blue (number 1) represents normal breast controls. Dark blue (number 2) are cancer samples. C) Overexpression in ovarian cancer (32 samples) from Welsh, et. al. [83]. P-value in T test 6.04-8 with a fold change of 23.955. Light blue (number 1) represents normal ovary controls. Dark blue (number 2) are cancer samples. D) Overexpression in teratomas from Korkola, et. al. [84] (20 samples). P-value in T test 6.64-10 with a fold change of 11.591. Light blue (number 1) represents normal testis controls. Dark blue (number 2) are cancer samples. E) Overexpression in embryonal carcinomas (21 samples) from Korkola, et. al. [84]. P-value in T test 2.22-6 with a fold change of 2.295. Light blue (number 1) represents normal testis controls. Dark blue (number 2) are cancer samples. [file 1476-4598-10-152-S2.JPEG]
